# Supplementary material for: SHOOT: phylogenetic gene search and ortholog inference
Source: Genome Biol. 2022 Mar 28;23:85. doi: 10.1186/s13059-022-02652-8 (PMC8962542; doi:10.1186/s13059-022-02652-8)
Supplement: Supplementary file 1 — Additional file 1. This file contains the supplemental figures and their associated legends. Table S1. UniProt 2020 Reference Proteomes - Species list. Table S2. Fungi - Species list. Table S3. Metazoan - species list. Table S4. Plants – species list. Table S5. Bacteria & Archaea - strains list [file 13059_2022_2652_MOESM1_ESM.docx]

**Supplementary Table 1: UniProt 2020 Reference Proteomes – Species list**

| **Domain** | **Species** | **Proteome ID** |
| --- | --- | --- |
| Archaea | Halobacterium salinarum | UP000000554 |
| Archaea | Korarchaeum cryptofilum | UP000001686 |
| Archaea | Methanocaldococcus jannaschii | UP000000805 |
| Archaea | Methanosarcina acetivorans | UP000002487 |
| Archaea | Nitrosopumilus maritimus | UP000000792 |
| Archaea | Saccharolobus solfataricus | UP000001974 |
| Archaea | Thermococcus kodakarensis | UP000000536 |
| Bacteria | Aquifex aeolicus | UP000000798 |
| Bacteria | Bacillus subtilis | UP000001570 |
| Bacteria | Bacteroides thetaiotaomicron | UP000001414 |
| Bacteria | Bradyrhizobium diazoefficiens | UP000002526 |
| Bacteria | Chlamydia trachomatis | UP000000431 |
| Bacteria | Chloroflexus aurantiacus | UP000002008 |
| Bacteria | Deinococcus radiodurans | UP000002524 |
| Bacteria | Dictyoglomus turgidum | UP000007719 |
| Bacteria | Escherichia coli | UP000000625 |
| Bacteria | Fusobacterium nucleatum | UP000002521 |
| Bacteria | Geobacter sulfurreducens | UP000000577 |
| Bacteria | Gloeobacter violaceus | UP000000557 |
| Bacteria | Helicobacter pylori | UP000000429 |
| Bacteria | Leptospira interrogans | UP000001408 |
| Bacteria | Mycobacterium tuberculosis | UP000001584 |
| Bacteria | Mycoplasma genitalium | UP000000807 |
| Bacteria | Neisseria meningitidis | UP000000425 |
| Bacteria | Pseudomonas aeruginosa | UP000002438 |
| Bacteria | Rhodopirellula baltica | UP000001025 |
| Bacteria | Streptomyces coelicolor | UP000001973 |
| Bacteria | Synechocystis sp. | UP000001425 |
| Bacteria | Thermodesulfovibrio yellowstonii | UP000000718 |
| Bacteria | Thermotoga maritima | UP000008183 |
| Eukaryota | Anopheles gambiae | UP000007062 |
| Eukaryota | Arabidopsis thaliana | UP000006548 |
| Eukaryota | Batrachochytrium dendrobatidis | UP000007241 |
| Eukaryota | Bos taurus | UP000009136 |
| Eukaryota | Branchiostoma floridae | UP000001554 |
| Eukaryota | Caenorhabditis elegans | UP000001940 |
| Eukaryota | Candida albicans | UP000000559 |
| Eukaryota | Canis lupus familiaris | UP000002254 |
| Eukaryota | Chlamydomonas reinhardtii | UP000006906 |
| Eukaryota | Ciona intestinalis | UP000008144 |
| Eukaryota | Cryptococcus neoformans | UP000002149 |
| Eukaryota | Danio rerio | UP000000437 |
| Eukaryota | Dictyostelium discoideum | UP000002195 |
| Eukaryota | Drosophila melanogaster | UP000000803 |
| Eukaryota | Gallus gallus | UP000000539 |
| Eukaryota | Giardia intestinalis | UP000001548 |
| Eukaryota | Gorilla gorilla gorilla | UP000001519 |
| Eukaryota | Helobdella robusta | UP000015101 |
| Eukaryota | Homo sapiens | UP000005640 |
| Eukaryota | Ixodes scapularis | UP000001555 |
| Eukaryota | Leishmania major | UP000000542 |
| Eukaryota | Lepisosteus oculatus | UP000018468 |
| Eukaryota | Monodelphis domestica | UP000002280 |
| Eukaryota | Monosiga brevicollis | UP000001357 |
| Eukaryota | Mus musculus | UP000000589 |
| Eukaryota | Nematostella vectensis | UP000001593 |
| Eukaryota | Neosartorya fumigata | UP000002530 |
| Eukaryota | Neurospora crassa | UP000001805 |
| Eukaryota | Oryza sativa subsp. japonica | UP000059680 |
| Eukaryota | Oryzias latipes | UP000001038 |
| Eukaryota | Pan troglodytes | UP000002277 |
| Eukaryota | Paramecium tetraurelia | UP000000600 |
| Eukaryota | Phaeosphaeria nodorum | UP000001055 |
| Eukaryota | Physcomitrella patens | UP000006727 |
| Eukaryota | Phytophthora ramorum | UP000005238 |
| Eukaryota | Plasmodium falciparum | UP000001450 |
| Eukaryota | Puccinia graminis | UP000008783 |
| Eukaryota | Rattus norvegicus | UP000002494 |
| Eukaryota | Saccharomyces cerevisiae | UP000002311 |
| Eukaryota | Schizosaccharomyces pombe | UP000002485 |
| Eukaryota | Sclerotinia sclerotiorum | UP000001312 |
| Eukaryota | Thalassiosira pseudonana | UP000001449 |
| Eukaryota | Tribolium castaneum | UP000007266 |
| Eukaryota | Trichomonas vaginalis | UP000001542 |
| Eukaryota | Ustilago maydis | UP000000561 |
| Eukaryota | Xenopus tropicalis | UP000008143 |
| Eukaryota | Yarrowia lipolytica | UP000001300 |
| Eukaryota | Zea mays | UP000007305 |

**Supplementary Table 1 Legend:**

The genomes used in the UniProt database, consisting of all genomes in the UniProt Reference Proteomes 2020 dataset.

**Supplementary Table 2: Fungi species list**

| **Species** | **Version** |
| --- | --- |
| *Agaricus bisporus* | var_burnettii_jb137_s8_gca_000300555.1 |
| *Amanita muscaria* | koide_bx008_gca_000827485.v1.0 |
| *Aspergillus fumigatus* | ASM1142v1 |
| *Aspergillus nidulans* | gca_002234985.ASM223498v1 |
| *Batrachochytrium salamandrivorans* | gca_002006685.V1 |
| *Blumeria graminis* | EF2 |
| *Botrytis cinerea* | ASM83294v1 |
| *Candida albicans* | gc75_gca_000773735.V1 |
| *Colletotrichum graminicola* | M1_001_V1 |
| *Cryptococcus neoformans* | ASM9104v1 |
| *Encephalitozoon intestinalis* | atcc_50506_gca_000146465.ASM14646v1 |
| *Enterocytozoon bieneusi* | h348_gca_000209485.ASM20948v1 |
| *Fusarium oxysporum* | FO2 |
| *Magnaporthe oryzae* | MG8 |
| *Mortierella elongata* | ag_77_gca_001651415.v2.0 |
| *Neurospora crassa* | NC12 |
| *Phaeosphaeria nodorum* | ASM14691v1 |
| *Puccinia graminis* | ASM14992v1 |
| *Rhizoctonia solani* | gca_001286725.AG22IIIB |
| *Rhizopus delemar* | ra_99_880_gca_000149305.RO3 |
| *Saccharomyces cerevisiae* | R64-1-1 |
| *Schizosaccharomyces pombe* | ASM294v2 |
| *Sclerotinia sclerotiorum* | ASM14694v1 |
| *Spizellomyces punctatus* | daom_br117_gca_000182565.V1 |
| *Ustilago maydis* | Umaydis521_2.0 |
| *Yarrowia lipolytica* | GCA_000002525.1 |
| *Zymoseptoria tritici* | MG2 |
| **Outgroup** |  |
| *Caenorhabditis elegans* | WBcel235 |
| *Dictyostelium discoideum* | dicty_2.7 |
| *Drosophila melanogaster* | BDGP6.32 |
| *Homo sapiens* | GRCh38 |
| *Monosiga brevicollis* | mx1_gca_000002865.V1.0 |

**Supplementary Table 2 Legend:**

The genomes used in the Fungi database, download from Ensembl.

**Supplementary Table 3: Metazoan species list**

| **Species** | **Source** | **Version** |
| --- | --- | --- |
| *Amphimedon queenslandica* | Metazoa 51 | Aqu1 |
| *Anolis carolinensis* | Ensembl 104 | v2 |
| *Anopheles gambiae* | Metazoa 51 | AgamP4 |
| *Apis mellifera* | Metazoa 51 | Amel_HAv3.1 |
| *Astatotilapia calliptera* | Ensembl 104 | fAstCal1.2 |
| *Bombyx mori* | Metazoa 51 | ASM15162v1 |
| *Bos taurus* | Ensembl 104 | ARS-UCD1.2 |
| *Branchiostoma lanceolatum* | Metazoa 51 | BraLan2 |
| *Bubo bubo* | Ensembl 104 | BubBub1.0 |
| *Caenorhabditis elegans* | Metazoa 51 | WBcel235 |
| *Callithrix jacchus* | Ensembl 104 | ASM275486v1 |
| *Callorhinchus milii* | Ensembl 104 | 6.1.3 |
| *Canis familiaris* | Ensembl 104 | CanFam3.1 |
| *Chrysemys picta* | Ensembl 104 | 3.0.3 |
| *Ciona intestinalis* | Ensembl 104 | KH |
| *Corvus moneduloides* | Ensembl 104 | UO_Cmon_1.0 |
| *Crocodylus porosus* | Ensembl 104 | comp1 |
| *Danio rerio* | Ensembl 104 | GRCz11 |
| *Daphnia magna* | Metazoa 51 | daphmag2.4 |
| *Drosophila melanogaster* | Metazoa 51 | BDGP6.32 |
| *Gadus morhua* | Ensembl 104 | gadMor3.0 |
| *Gallus gallus* | Ensembl 104 | GRCg6a |
| *Glossina morsitans* | Metazoa 51 | GmorY1 |
| *Helobdella robusta* | Metazoa 51 | Helro1 |
| *Homo sapiens* | Ensembl 104 | GRCh38 |
| *Ixodes scapularis* | Metazoa 51 | IscaW1 |
| *Latimeria chalumnae* | Ensembl 104 | LatCha1 |
| *Lepisosteus oculatus* | Ensembl 104 | LepOcu1 |
| *Leptobrachium leishanense* | Ensembl 104 | ASM966780v1 |
| *Mnemiopsis leidyi* | Metazoa 51 | MneLei_Aug2011 |
| *Monodelphis domestica* | Ensembl 104 | ASM229v1 |
| *Mus musculus* | Ensembl 104 | GRCm39 |
| *Nematostella vectensis* | Metazoa 51 | ASM20922v1 |
| *Octopus bimaculoides* | Ensembl 104 | PRJNA270931 |
| *Oncorhynchus mykiss* | Ensembl 104 | Omyk_1.0 |
| *Ornithorhynchus anatinus* | Ensembl 104 | mOrnAna1.p.v1 |
| *Oryzias latipes* | Ensembl 104 | ASM223467v1 |
| *Pan troglodytes* | Ensembl 104 | Pan_tro_3.0 |
| *Petromyzon marinus* | Ensembl 104 | Pmarinus_7.0 |
| *Phascolarctos cinereus* | Ensembl 104 | unsw_v4.1 |
| *Poecilia formosa* | Ensembl 104 | 5.1.2 |
| *Rattus norvegicus* | Ensembl 104 | Rnor_6.0 |
| *Schistosoma mansoni* | Metazoa 51 | ASM23792v2 |
| *Strongylocentrotus purpuratus* | Metazoa 51 | Spur_5.0 |
| *Tetraodon nigroviridis* | Ensembl 104 | TETRAODON8 |
| *Thelohanellus kitauei* | Metazoa 51 | ASM82789v1 |
| *Trichinella spiralis* | Metazoa 51 | Tspiralis1 |
| *Trichoplax adhaerens* | Metazoa 51 | ASM15027v1 |
| *Xenopus tropicalis* | Ensembl 104 | v9.1 |

**Supplementary Table 3 Legend:**

The genomes used in the Metazoa database.

**Supplementary Table 4: Plants species list**

| **Species** | **Source** | **Version** |
| --- | --- | --- |
| *Amborella trichopoda* | Phytozome | v1.0 |
| *Anthoceros punctatus* | hornworts.uzh.ch | Apun |
| *Aquilegia coerulea* | Phytozome | v3.1 |
| *Arabidopsis thaliana* | Phytozome | TAIR10 |
| *Azolla filiculoides* | fernbase | Azfi |
| *Brassica oleracea* | Phytozome | v1.0 |
| *Chara braunii* | Ensembl | Cbr_1.0 |
| *Chlamydomonas reinhardtii* | Phytozome | v5.5 |
| *Eucalyptus grandis* | Phytozome | v2.0 |
| *Gingko biloba* | Plaza | gbi |
| *Glycine max* | Phytozome | a2.v1 |
| *Gossypium raimondii* | Phytozome | v2.1 |
| *Hordeum vulgare* | Phytozome | r1 |
| *Manihot esculenta* | Phytozome | v6.1 |
| *Marchantia polymorpha* | Phytozome | v3.1 |
| *Micromonas spRCC299* | Phytozome | v3.0 |
| *Musa acuminata* | Phytozome | v1.0 |
| *Oryza sativa* | Phytozome | v7_JGI |
| *Ostreococcus lucimarinus* | Phytozome | v2.0 |
| *Physcomitrella patens* | Phytozome | v3.3 |
| *Picea glauca* | Plaza | pgl |
| *Pinus sylvestris* | Plaza | psy |
| *Prunus persica* | Phytozome | v2.1 |
| *Selaginella moellendorffii* | Phytozome | v1.0 |
| *Setaria italica* | Phytozome | v2.2 |
| *Solanum lycopersicum* | Phytozome | iTAG2.4 |
| *Spirodela polyrhiza* | Phytozome | v2 |
| *Triticum aestivum* | Phytozome | v2.2 |
| *Volvox carteri* | Phytozome | v2.1 |
| *Zea mays* | Phytozome | PH207 v1.1 |
|  |  |  |
| **Outgroup** |  |  |
| Chondrus crispus | Ensembl | ASM35022v2 |
| Cyanidioschyzon merolae | Ensembl | ASM9120v1 |
| Galdieria sulphuraria | Ensembl | ASM34128v1 |

**Supplementary Table 4 Legend:**

The genomes used in the Plants database.

**Supplementary Table 5: Bacterial & Archaeal strains list**

| **UniProt proteome** | **NCBI taxon** | **Name in SHOOT** | **Selection** |
| --- | --- | --- | --- |
| UP000000425 | 122586 | Neisseria_meningitidis | QfO UniProt ref. prot. |
| UP000000429 | 85962 | Helicobacter_pylori | QfO UniProt ref. prot. |
| UP000000431 | 272561 | Chlamydia_trachomatis | QfO UniProt ref. prot. |
| UP000000536 | 69014 | Thermococcus_kodakarensis | QfO UniProt ref. prot. |
| UP000000554 | 64091 | Halobacterium_salinarum | QfO UniProt ref. prot. |
| UP000000557 | 251221 | Gloeobacter_violaceus | QfO UniProt ref. prot. |
| UP000000577 | 243231 | Geobacter_sulfurreducens | QfO UniProt ref. prot. |
| UP000000625 | 83333 | Escherichia_coli | QfO UniProt ref. prot. |
| UP000000718 | 289376 | Thermodesulfovibrio_yellowstonii | QfO UniProt ref. prot. |
| UP000000792 | 436308 | Nitrosopumilus_maritimus | QfO UniProt ref. prot. |
| UP000000798 | 224324 | Aquifex_aeolicus | QfO UniProt ref. prot. |
| UP000000805 | 243232 | Methanocaldococcus_jannaschii | QfO UniProt ref. prot. |
| UP000000807 | 243273 | Mycoplasma_genitalium | QfO UniProt ref. prot. |
| UP000001025 | 243090 | Rhodopirellula_baltica | QfO UniProt ref. prot. |
| UP000001408 | 189518 | Leptospira_interrogans | QfO UniProt ref. prot. |
| UP000001414 | 226186 | Bacteroides_thetaiotaomicron | QfO UniProt ref. prot. |
| UP000001425 | 1111708 | Synechocystis_Kazusa | QfO UniProt ref. prot. |
| UP000001570 | 224308 | Bacillus_subtilis | QfO UniProt ref. prot. |
| UP000001584 | 83332 | Mycobacterium_tuberculosis | QfO UniProt ref. prot. |
| UP000001686 | 374847 | Korarchaeum_cryptofilum | QfO UniProt ref. prot. |
| UP000001973 | 100226 | Streptomyces_coelicolor | QfO UniProt ref. prot. |
| UP000001974 | 273057 | Saccharolobus_solfataricus | QfO UniProt ref. prot. |
| UP000002008 | 324602 | Chloroflexus_aurantiacus | QfO UniProt ref. prot. |
| UP000002438 | 208964 | Pseudomonas_aeruginosa | QfO UniProt ref. prot. |
| UP000002487 | 188937 | Methanosarcina_acetivorans | QfO UniProt ref. prot. |
| UP000002521 | 190304 | Fusobacterium_nucleatum | QfO UniProt ref. prot. |
| UP000002524 | 243230 | Deinococcus_radiodurans | QfO UniProt ref. prot. |
| UP000002526 | 224911 | Bradyrhizobium_diazoefficiens | QfO UniProt ref. prot. |
| UP000007719 | 515635 | Dictyoglomus_turgidum | QfO UniProt ref. prot. |
| UP000008183 | 243274 | Thermotoga_maritima | QfO UniProt ref. prot. |
| UP000000265 | 272620 | Klebsiella_pneumoniae | Highly cited |
| UP000000579 | 71421 | Haemophilus_influenzae | Highly cited |
| UP000000580 | 262316 | Mycolicibacterium_paratuberculosis | Highly cited |
| UP000000584 | 243277 | Vibrio_cholerae | Highly cited |
| UP000000586 | 171101 | Streptococcus_pneumoniae | Highly cited |
| UP000000588 | 242619 | Porphyromonas_gingivalis | Highly cited |
| UP000000609 | 272624 | Legionella_pneumophila | Highly cited |
| UP000000799 | 192222 | Campylobacter_jejuni | Highly cited |
| UP000000813 | 176299 | Agrobacterium_fabrum | Highly cited |
| UP000000815 | 632 | Yersinia_pestis | Highly cited |
| UP000000817 | 169963 | Listeria_monocytogenes | Highly cited |
| UP000000818 | 195102 | Clostridium_perfringens | Highly cited |
| UP000001006 | 623 | Shigella_flexneri | Highly cited |
| UP000001014 | 99287 | Salmonella_typhimurium | Highly cited |
| UP000001978 | 272563 | Clostridioides_difficile | Highly cited |
| UP000002196 | 272623 | Lactococcus_lactis | Highly cited |
| UP000002256 | 395491 | Rhizobium_leguminosarum | Highly cited |
| UP000006381 | 272621 | Lactobacillus_acidophilus | Highly cited |
| UP000007477 | 871585 | Acinetobacter_calcoaceticus | Highly cited |
| UP000008319 | 529507 | Proteus_mirabilis | Highly cited |
| UP000008816 | 93061 | Staphylococcus_aureus | Highly cited |
| UP000014594 | 1260356 | Enterococcus_faecalis | Highly cited |
| UP000075229 | 140 | Borrelia_hermsii | Highly cited |
| UP000198289 | 615 | Serratia_marcescens | Highly cited |
| UP000028936 | 1528098 | Rickettsiales_bacterium | Mitochondrion relative |
| UP000180235 | 1188229 | Gloeomargarita_lithophora | Chloroplast relative |
| UP000000543 | 279808 | Staphylococcus_haemolyticus | Phylo. sampling |
| UP000000547 | 167879 | Colwellia_psychrerythraea | Phylo. sampling |
| UP000000645 | 232721 | Acidovorax_JS42 | Phylo. sampling |
| UP000001169 | 272569 | Haloarcula_marismortui | Phylo. sampling |
| UP000001361 | 883 | Desulfovibrio_vulgaris | Phylo. sampling |
| UP000001362 | 243159 | Acidithiobacillus_ferrooxidans | Phylo. sampling |
| UP000001961 | 64471 | Synechococcus_CC9311 | Phylo. sampling |
| UP000002011 | 471854 | Dyadobacter_fermentans | Phylo. sampling |
| UP000002139 | 448385 | Sorangium_cellulosum | Phylo. sampling |
| UP000002145 | 203119 | Hungateiclostridium_thermocellum | Phylo. sampling |
| UP000002148 | 388919 | Streptococcus_sanguinis | Phylo. sampling |
| UP000002208 | 546414 | Deinococcus_deserti | Phylo. sampling |
| UP000002257 | 395965 | Methylocella_silvestris | Phylo. sampling |
| UP000002386 | 471223 | Geobacillus_WCH70 | Phylo. sampling |
| UP000002457 | 521011 | Methanosphaerula_palustris | Phylo. sampling |
| UP000002495 | 235279 | Helicobacter_hepaticus | Phylo. sampling |
| UP000003277 | 742743 | Dialister_succinatiphilus | Phylo. sampling |
| UP000003415 | 469616 | Fusobacterium_mortiferum | Phylo. sampling |
| UP000003446 | 661087 | Olsenella_F0356 | Phylo. sampling |
| UP000003855 | 665956 | Subdoligranulum_4-3-54A2FAA | Phylo. sampling |
| UP000003981 | 621372 | Paenibacillus_D14 | Phylo. sampling |
| UP000004073 | 1105031 | Clostridium_MSTE9 | Phylo. sampling |
| UP000004090 | 428127 | Absiella_dolichum | Phylo. sampling |
| UP000004259 | 246199 | Ruminococcus_albus | Phylo. sampling |
| UP000004478 | 1225176 | Cecembia_lonarensis | Phylo. sampling |
| UP000004870 | 638300 | Cardiobacterium_hominis | Phylo. sampling |
| UP000005262 | 768704 | Desulfosporosinus_meridiei | Phylo. sampling |
| UP000006229 | 1131455 | Mycoplasma_canis | Phylo. sampling |
| UP000006415 | 857290 | Scardovia_wiggsiae | Phylo. sampling |
| UP000006556 | 370438 | Pelotomaculum_thermopropionicum | Phylo. sampling |
| UP000006743 | 557723 | Haemophilus_parasuis | Phylo. sampling |
| UP000007271 | 1185325 | Lactobacillus_coryniformis | Phylo. sampling |
| UP000007753 | 452662 | Sphingobium_japonicum | Phylo. sampling |
| UP000007995 | 997888 | Bacteroides_finegoldii | Phylo. sampling |
| UP000008204 | 41431 | Rippkaea_orientalis | Phylo. sampling |
| UP000008212 | 243275 | Treponema_denticola | Phylo. sampling |
| UP000008308 | 263358 | Micromonospora_maris | Phylo. sampling |
| UP000008701 | 290317 | Chlorobium_phaeobacteroides | Phylo. sampling |
| UP000009044 | 634177 | Komagataeibacter_medellinensis | Phylo. sampling |
| UP000009154 | 1112204 | Gordonia_polyisoprenivorans | Phylo. sampling |
| UP000011615 | 1230457 | Haloterrigena_limicola | Phylo. sampling |
| UP000011728 | 931276 | Clostridium_saccharoperbutylacetonicum | Phylo. sampling |
| UP000013232 | 1123367 | Thauera_linaloolentis | Phylo. sampling |
| UP000017993 | 1262970 | Subdoligranulum_CAG314 | Phylo. sampling |
| UP000018014 | 1262708 | Bacillus_CAG988 | Phylo. sampling |
| UP000018042 | 1262875 | Eggerthella_CAG209 | Phylo. sampling |
| UP000018237 | 1262989 | Firmicutes_bacterium | Phylo. sampling |
| UP000018329 | 1262693 | Alistipes_CAG268 | Phylo. sampling |
| UP000018361 | 1263102 | Prevotella_copri | Phylo. sampling |
| UP000018415 | 1341679 | Acinetobacter_indicus | Phylo. sampling |
| UP000019028 | 1239307 | Sodalis_praecaptivus | Phylo. sampling |
| UP000019082 | 1302241 | Cutibacterium_acnes | Phylo. sampling |
| UP000019222 | 1224164 | Corynebacterium_vitaeruminis | Phylo. sampling |
| UP000019267 | 1276246 | Spiroplasma_culicicola | Phylo. sampling |
| UP000020878 | 1454005 | Candidatus_Accumulibacter | Phylo. sampling |
| UP000028780 | 156978 | Corynebacterium_imitans | Phylo. sampling |
| UP000028875 | 1462526 | Virgibacillus_massiliensis | Phylo. sampling |
| UP000029622 | 1156417 | Caloranaerobacter_azorensis | Phylo. sampling |
| UP000030960 | 561184 | Mameliella_alba | Phylo. sampling |
| UP000031057 | 1348853 | Novosphingobium_malaysiense | Phylo. sampling |
| UP000031627 | 1410383 | Candidatus_Tachikawaea | Phylo. sampling |
| UP000032279 | 1335616 | Paucilactobacillus_wasatchensis | Phylo. sampling |
| UP000032287 | 137591 | Weissella_cibaria | Phylo. sampling |
| UP000033511 | 43662 | Pseudoalteromonas_piscicida | Phylo. sampling |
| UP000036114 | 1628212 | Chromobacterium_LK11 | Phylo. sampling |
| UP000036921 | 1581033 | Bacillus_FJAT-21945 | Phylo. sampling |
| UP000037530 | 171383 | Vibrio_hepatarius | Phylo. sampling |
| UP000037870 | 1592329 | Actinobacteria_bacterium | Phylo. sampling |
| UP000044377 | 1109412 | Brenneria_goodwinii | Phylo. sampling |
| UP000050971 | 1736540 | Aeromicrobium_Root472D3 | Phylo. sampling |
| UP000051467 | 1736232 | Arthrobacter_Leaf69 | Phylo. sampling |
| UP000051585 | 1736381 | Aureimonas_Leaf454 | Phylo. sampling |
| UP000051643 | 270918 | Salegentibacter_mishustinae | Phylo. sampling |
| UP000051802 | 676599 | Stenotrophomonas_panacihumi | Phylo. sampling |
| UP000053086 | 1700846 | Lysinibacillus_F5 | Phylo. sampling |
| UP000054024 | 146536 | Streptomyces_curacoi | Phylo. sampling |
| UP000054457 | 1685377 | Microbulbifer_ZGT114 | Phylo. sampling |
| UP000057134 | 1766 | Mycolicibacterium_fortuitum | Phylo. sampling |
| UP000058305 | 412690 | Microterricola_viridarii | Phylo. sampling |
| UP000061489 | 1420916 | Marinobacter_similis | Phylo. sampling |
| UP000065824 | 1702325 | Chelatococcus_CO-6 | Phylo. sampling |
| UP000070463 | 1698267 | Candidate_MSBL1-archaeon | Phylo. sampling |
| UP000077018 | 683316 | Frankia_EI5c | Phylo. sampling |
| UP000077275 | 47311 | Methanobrevibacter_cuticularis | Phylo. sampling |
| UP000077319 | 1822215 | Erythrobacter_HI00D59 | Phylo. sampling |
| UP000093220 | 189873 | Bradyrhizobium_LMTRsp-3 | Phylo. sampling |
| UP000093585 | 319501 | Brevibacillus_WF146 | Phylo. sampling |
| UP000094329 | 1891921 | Piscirickettsia_litoralis | Phylo. sampling |
| UP000094487 | 1888892 | Sphingomonas_turrisvirgatae | Phylo. sampling |
| UP000094689 | 1842539 | Bosea_RAC05 | Phylo. sampling |
| UP000095256 | 762845 | Enterococcus_rivorum | Phylo. sampling |
| UP000176615 | 1739315 | Globicatella_HMSC072A10 | Phylo. sampling |
| UP000182624 | 43305 | Butyrivibrio_proteoclasticus | Phylo. sampling |
| UP000184455 | 1855338 | Nitrosospira_Nsp11 | Phylo. sampling |
| UP000184520 | 634436 | Marisediminitalea_aggregata | Phylo. sampling |
| UP000186096 | 58117 | Microbispora_rosea | Phylo. sampling |
| UP000186602 | 1261634 | Roseburia_sp499 | Phylo. sampling |
| UP000187327 | 1883416 | Halomonas_sp1513 | Phylo. sampling |
| UP000187995 | 1805827 | Rhodococcus_MTM3W5 | Phylo. sampling |
| UP000190286 | 745368 | Gemmiger_formicilis | Phylo. sampling |
| UP000191905 | 1873176 | Pseudaminobacter_manganicus | Phylo. sampling |
| UP000192042 | 1325564 | Nitrospira_japonica | Phylo. sampling |
| UP000193006 | 199441 | Alkalihalobacillus_krulwichiae | Phylo. sampling |
| UP000193136 | 1969733 | Geothermobacter_EPR-M | Phylo. sampling |
| UP000194216 | 1985172 | Sphingomonas_IBVSS2 | Phylo. sampling |
| UP000194221 | 1635173 | Tenacibaculum_holothuriorum | Phylo. sampling |
| UP000195076 | 1932621 | Nostoc_T09 | Phylo. sampling |
| UP000195161 | 1929267 | Flavobacterium_FPG59 | Phylo. sampling |
| UP000195529 | 1965622 | Megasphaera_An286 | Phylo. sampling |
| UP000195781 | 1232426 | Collinsella_massiliensis | Phylo. sampling |
| UP000197446 | 431059 | Pelomonas_puraquae | Phylo. sampling |
| UP000198589 | 1798228 | Blastococcus_DSMsp-46838 | Phylo. sampling |
| UP000198953 | 46177 | Nonomuraea_pusilla | Phylo. sampling |
| UP000199067 | 1780377 | Coriobacteriaceae_bacterium | Phylo. sampling |
| UP000199242 | 1141221 | Chryseobacterium_taihuense | Phylo. sampling |
| UP000199432 | 1882749 | Opitutus_GAS368 | Phylo. sampling |
| UP000199671 | 332524 | Actinomyces_ruminicola | Phylo. sampling |
| UP000199705 | 551996 | Mucilaginibacter_gossypii | Phylo. sampling |
| UP000199768 | 1881066 | Phyllobacterium_YR620 | Phylo. sampling |
| UP000199802 | 1965654 | Lachnoclostridium_An76 | Phylo. sampling |
| UP000202922 | 1524263 | Confluentimicrobium_lipolyticum | Phylo. sampling |
| UP000215509 | 554312 | Paenibacillus_rigui | Phylo. sampling |
| UP000216308 | 1383851 | Halorubrum_halodurans | Phylo. sampling |
| UP000217076 | 83401 | Roseospirillum_parvum | Phylo. sampling |
| UP000217289 | 1294270 | Melittangium_boletus | Phylo. sampling |
| UP000221394 | 442709 | Flavimobilis_soli | Phylo. sampling |
| UP000222106 | 638953 | Georgenia_soli | Phylo. sampling |
| UP000230810 | 2049589 | Pseudomonas_HLS-6 | Phylo. sampling |
| UP000232878 | 2058137 | Polaribacter_ALD11 | Phylo. sampling |
| UP000232889 | 1250229 | Ulvibacter_MAR-2010-11 | Phylo. sampling |
| UP000235352 | 2029108 | Bacillus_UMB0899 | Phylo. sampling |
| UP000236356 | 2067550 | Clostridium_chh4-2 | Phylo. sampling |
| UP000236731 | 797291 | Sphingobacterium_lactis | Phylo. sampling |
| UP000238164 | 75385 | Micropruina_glycogenica | Phylo. sampling |
| UP000238375 | 1469603 | Spirosoma_oryzae | Phylo. sampling |
| UP000243063 | 1245526 | Pseudomonas_guangdongensis | Phylo. sampling |
| UP000243494 | 2020948 | Romboutsia_maritimum | Phylo. sampling |
| UP000244224 | 589035 | Gemmobacter_caeni | Phylo. sampling |
| UP000245108 | 2108523 | Lawsonibacter_asaccharolyticus | Phylo. sampling |
| UP000245507 | 2201891 | Nocardioides_silvaticus | Phylo. sampling |
| UP000245623 | 2173179 | Microbacterium_4-13 | Phylo. sampling |
| UP000245926 | 2202825 | Methylobacterium_durans | Phylo. sampling |
| UP000247832 | 670078 | Arthrobacter_livingstonensis | Phylo. sampling |
| UP000249065 | 2230885 | Roseicella_frigidaeris | Phylo. sampling |
| UP000250434 | 1804986 | Amycolatopsis_albispora | Phylo. sampling |
| UP000252733 | 989 | Marinilabilia_salmonicolor | Phylo. sampling |
| UP000253318 | 1931232 | Marinitenerispora_sediminis | Phylo. sampling |
| UP000254875 | 2211104 | Paraburkholderia_lacunae | Phylo. sampling |
| UP000260665 | 2184758 | Rhodoferax_IMCC26218 | Phylo. sampling |
| UP000265971 | 1825976 | Neorhizobium_NCHU2750 | Phylo. sampling |
| UP000266860 | 1630648 | Novosphingobium_MD-1 | Phylo. sampling |
| UP000269803 | 2485200 | Frondihabitans_PhB188 | Phylo. sampling |
| UP000273083 | 1329262 | Mobilisporobacter_senegalensis | Phylo. sampling |
| UP000275325 | 2495580 | Sphingomonas_TF3 | Phylo. sampling |
| UP000276437 | 1930071 | Methylomusa_anaerophila | Phylo. sampling |
| UP000279089 | 1647451 | Chitinophaga_barathri | Phylo. sampling |
| UP000282084 | 2072 | Saccharothrix_australiensis | Phylo. sampling |
| UP000287188 | 2014872 | Dictyobacter_kobayashii | Phylo. sampling |
| UP000287890 | 2507159 | Clostridium_JN-9 | Phylo. sampling |
| UP000288096 | 45657 | Desulfonema_ishimotonii | Phylo. sampling |
| UP000288291 | 2495899 | Lactobacillus_xujianguonis | Phylo. sampling |
| UP000288967 | 2501295 | Dyella_M7H15-1 | Phylo. sampling |
| UP000289784 | 2137479 | Pseudoxanthomonas_composti | Phylo. sampling |
| UP000292120 | 2528630 | Aquabacterium_KMB7 | Phylo. sampling |
| UP000294096 | 2510646 | Loktanella_IMCC34160 | Phylo. sampling |
| UP000294498 | 1539049 | Dinghuibacter_silviterrae | Phylo. sampling |
| UP000295707 | 1537524 | Thiogranum_longum | Phylo. sampling |
| UP000297351 | 2561925 | Brevundimonas_S30B | Phylo. sampling |
| UP000306069 | 2040651 | Campylobacter_12-5580 | Phylo. sampling |
| UP000307244 | 2571272 | Pedobacter_RP-3-15 | Phylo. sampling |
| UP000307467 | 343240 | Thiotrophic_endosymbiont | Phylo. sampling |
| UP000307507 | 2565924 | Flavobacterium_CC-CTC003 | Phylo. sampling |
| UP000307657 | 2565367 | Lacinutrix_CAUsp-1491 | Phylo. sampling |
| UP000315440 | 2527991 | Pseudobythopirellula_maris | Phylo. sampling |
| UP000316225 | 384678 | Paracoccus_sulfuroxidans | Phylo. sampling |
| UP000316304 | 2528004 | Novipirellula_galeiformis | Phylo. sampling |
| UP000318165 | 92402 | Mycoplasma_equirhinis | Phylo. sampling |
| UP000318431 | 1036180 | Massilia_lurida | Phylo. sampling |
| UP000318566 | 2768454 | Streptomyces_SLBN-118 | Phylo. sampling |
| UP000319173 | 713054 | TM7_phylum | Phylo. sampling |
| UP000322791 | 2606448 | Hymenobacter_KIGAM108 | Phylo. sampling |
| UP000324880 | 1948890 | Rhodobacterales_bacterium | Phylo. sampling |
| UP000325372 | 2613842 | Wenzhouxiangella_W260 | Phylo. sampling |
| UP000326711 | 2487892 | Corynebacterium_LMM-1652 | Phylo. sampling |
| UP000326944 | 2590022 | Sulfurimonas_GYSZ1 | Phylo. sampling |
| UP000437955 | 2653936 | Tetrasphaera_F2B08 | Phylo. sampling |
| UP000441772 | 2650774 | Bifidobacterium_LMGsp-31471 | Phylo. sampling |
| UP000462055 | 2650748 | Actinomadura_LD22 | Phylo. sampling |
| UP000474632 | 2710884 | Parapusillimonas_SGNA-6 | Phylo. sampling |
| UP000476210 | 343235 | Methanotrophic_endosymbiont | Phylo. sampling |
| UP000477884 | 2703788 | Edaphobacter_12200R-103 | Phylo. sampling |
| UP000481552 | 2706104 | Streptomyces_SID8455 | Phylo. sampling |
| UP000500686 | 754515 | Mycoplasma_ES2806-GEN | Phylo. sampling |
| UP000502894 | 2708020 | Legionella_TUM19329 | Phylo. sampling |
| UP000503441 | 2714933 | Leucobacter_HDW9A | Phylo. sampling |
| UP000505377 | 2736640 | Pseudonocardia_broussonetiae | Phylo. sampling |

**Supplementary Table 5 Legend:**

The genomes used in the Bacteria & Archaea database, downloaded from UniProt on 21-08-20.

# Supplementary Figures

**Figure S1**

**
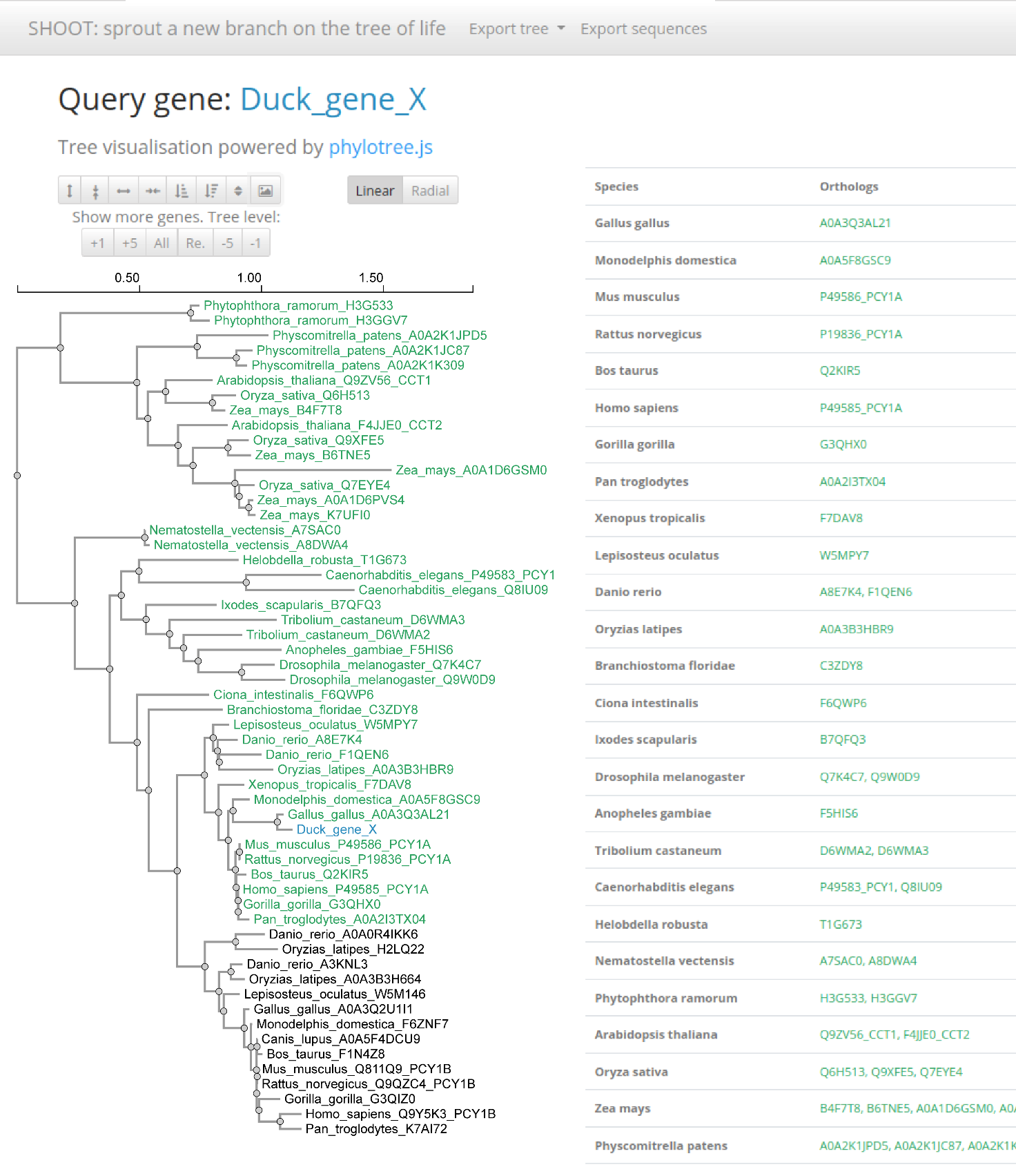
**

**Figure S2**

**
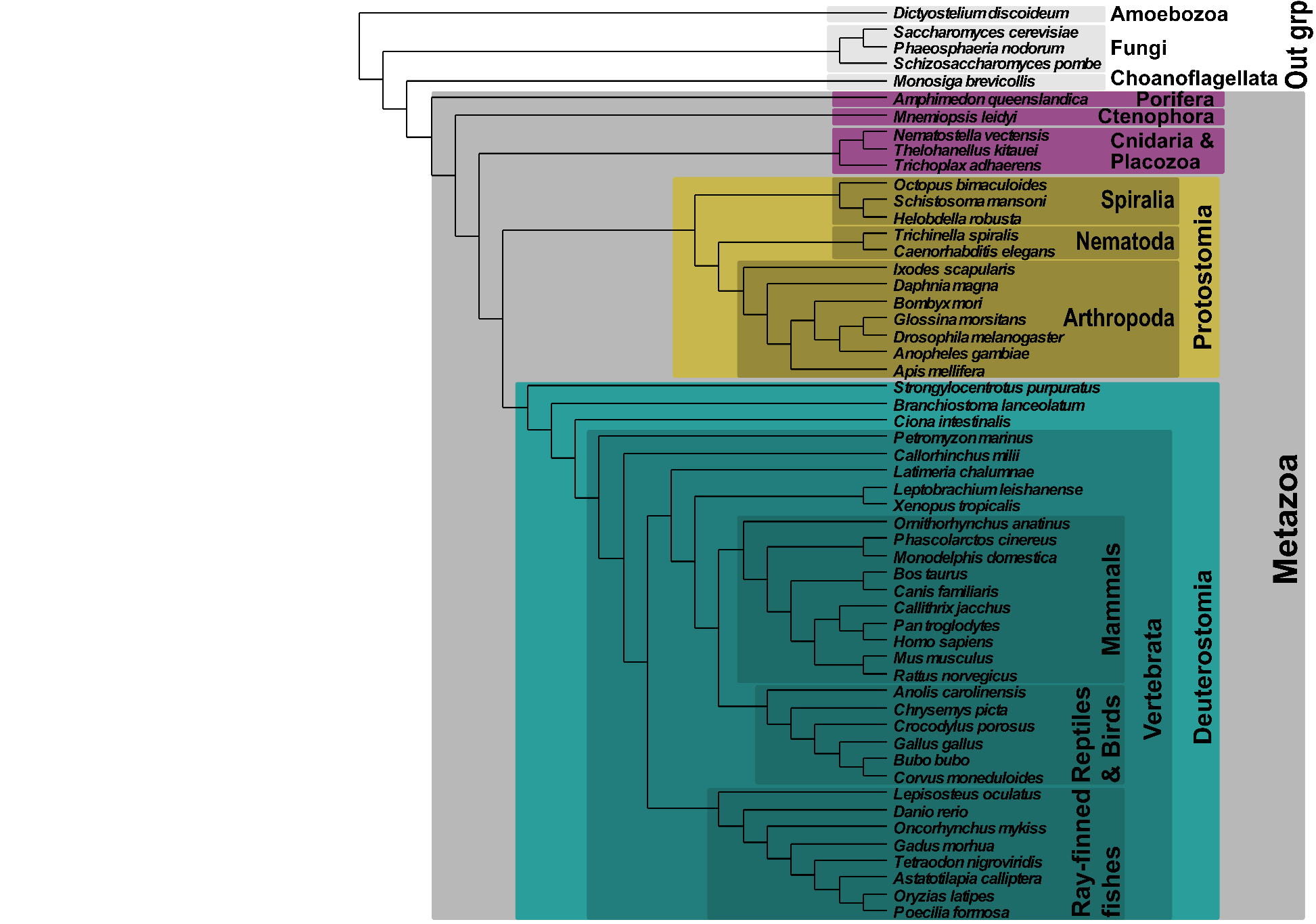
**

## Supplementary Figure Legends

**Figure S1.** An example gene tree and orthologs table returned by SHOOT. Here, the UniProt Reference Proteomes database was searched using a for a query gene sequence labelled “Duck_gene_X”. This corresponds to the Duck protein ENSAPLP00000002788, which is not included in the database.

**Figure S2.** Phylogeny for the species in the Metazoan dataset.
