# Supplementary material for: SHOOT: phylogenetic gene search and ortholog inference
Source: Genome Biol. 2022 Mar 28;23:85. doi: 10.1186/s13059-022-02652-8 (PMC8962542; doi:10.1186/s13059-022-02652-8)
Supplement: Supplementary file 2 — Additional file 2. Review history. [file 13059_2022_2652_MOESM2_ESM.docx]

Review History

**First round of review**

**Reviewer 1**

**Were you able to assess all statistics in the manuscript, including the appropriateness of statistical tests used?** No.

**Were you able to directly test the methods?** Yes.

**Comments to author:**

The authors implement the SHOOT web server that lets users search a protein sequence and get back a maximum likelihood gene tree for the sequence. The tool utilies a set of well-established tools including DIAMOND, OrthoFinder, MAFF, IQ-TREE and EPA-ng, etc. It is very convinent and a time saver for searching ortholog.

The tool use EPA-ng to place a query gene to a subtree and then grafted it back to the original larger tree using supertree method. It would be better to proof or verify that this method is equivalent to place the gene to the orginal larger tree directly.

The web interface is very clean and easy to use. However, there are some parameters of the tools internally used that may have high impact on the result. It would be better to provide these parameters so that users can fine-tune these paramters to explore the results.

In addition to bootstrap values labled on the tree, the uncertainty of the placement (either branch length or place position) is alos of interest. Can you incoporate such information on the tree?

Figure 4 metioned on the manuscript is missing.

**Reviewer 2**

**Were you able to assess all statistics in the manuscript, including the appropriateness of statistical tests used?** Yes. All presented results are statistically sound.

**Were you able to directly test the methods?** Yes.

**Comments to author:**

This manuscript presents SHOOT, a phylogenetic search method that allows searching orthologs for a user-defined query sequence against a reference database. Starting from a profile search against pre-computed homology groups in the reference database using DIAMOND, fast phylogenetic tree inference is applied to add the query sequence to the homology group phylogenetic tree using MAFFT. The authors implemented various speed optimizations (e.g. building a profile using a set of representative sequences instead of all sequence within a homologous group) resulting in a tool that is almost as fast as BLAST.
Although the developed approach is not completely novel and extends previously developed methods (which the authors correctly cite), this is (again) a versatile, efficient and state-of-the-art tool developed by the Kelley lab. Overall, the presentation of the methodology is clear and the benchmarks clearly show the high accuracy and strength of this method. Both a web-version and source code are available offering access to biologist and data scientists. Below a set of mainly minor comments.

Comments
Page 2, line 36. This paragraph ends with FASTA and BLAST, why are DIAMOND and MMSEQS2 not mentioned here? Currently, this paragraph is not giving an up-to-date overview of the important sequence similarity methods used in bioinformatics.

Page 2, line 47: in case tools like eggNOG-mapper (https://pubmed.ncbi.nlm.nih.gov/34597405/ ) or TRAPID (https://pubmed.ncbi.nlm.nih.gov/34197621/) offer similar functionality, please add these and/or explain how SHOOT is different.

Page 3, line 71. When searching a sequence against the pre-computed homology groups, is there always a clear top-match, with superior score to the second-best homology group? What happens in case the two top matches have identical scores?

(major) Page 5 and Figure 1: how does the speed and accuracy of SHOOT compare to a DIAMOND best-hit approach?

Output SHOOT: a tab-delimited "species /t gene_id /t sequence" output file would be useful to integrate SHOOT in data analysis workflows (when running locally). Is this possible? A related minor comment, did the authors consider offering SHOOT through an API?

Tool and website (www.shoot.bio). The website does not mention under which license this tool is released. Furthermore, the Methods section mentions the source code is available (via git) to run this on local hardware, please add this info to the SHOOT website (FAQ).

Page 9, line 208 "While, SHOOT identifies" rephrase

Page 10, line 244 "The advantage of the creating complete homologous groups" rephrase

Suppl. Info: Several of the tables lack (Uniprot proteome) identifiers to indicate which genome annotation was used. Please add this info so users know which genome assembly/annotation version is integrated in the reference databases.

**Authors Response**

**Point-by-point responses to the reviewers’ comments:**

Reviewer 1

Comment 1
The authors implement the SHOOT web server that lets users search a protein sequence and get back a maximum likelihood gene tree for the sequence. The tool utilies a set of well-established tools including DIAMOND, OrthoFinder, MAFF, IQ-TREE and EPA-ng, etc. It is very convinent and a time saver for searching ortholog. The tool use EPA-ng to place a query gene to a subtree and then grafted it back to the original larger tree using supertree method. It would be better to proof or verify that this method is equivalent to place the gene to the orginal larger tree directly.

Response 1
We thank the Reviewer for raising this point. We have performed the requested analysis, with the new results now shown in a new main manuscript figure, Figure 4. A description of the results has also been added to the text. To further address this issue we have also provided a new option for the SHOOT command line tool and web server to allow a user to choose between these two methods (note, this supertree method only affects assignments to the very largest trees in the database accounting for at most 40 trees of a database). We have stated this option in the manuscript.

Comment 2
The web interface is very clean and easy to use. However, there are some parameters of the tools internally used that may have high impact on the result. It would be better to provide these parameters so that users can fine-tune these paramters to explore the results.

Response 2
We thank the reviewer for their supportive comments! We have now added options to the web & command line interfaces to allow users to control the adjustable settings of the algorithm: Whether to use the supertree method or use phylogenetic methods of the full tree (for those cases affected); DIAMOND sensitivity setting; MAFFT settings used by sHOOT that affect accuracy/runtime; whether to use the DIAMOND “profiles” database or the full set of sequences.
In addition to bootstrap values labled on the tree, the uncertainty of the placement (either branch length or place position) is alos of interest. Can you incoporate such information on the tree?

Response 3
We thank the reviewer for this suggestion, we have now incorporated the likelihood ratio weight for the placement of the query gene in the tree. The branch length for the placement of the gene in the tree is already applied.

Comment 4
Figure 4 metioned on the manuscript is missing.

Response 4
This was a typo that we’ve now fixed.

Reviewer 2
Comment 1
This manuscript presents SHOOT, a phylogenetic search method that allows searching orthologs for a user-defined query sequence against a reference database. Starting from a profile search against pre-computed homology groups in the reference database using DIAMOND, fast phylogenetic tree inference is applied to add the query sequence to the homology group phylogenetic tree using MAFFT. The authors implemented various speed optimizations (e.g. building a profile using a set of representative sequences instead of all sequence within a homologous group) resulting in a tool that is almost as fast as BLAST.
Although the developed approach is not completely novel and extends previously developed methods (which the authors correctly cite), this is (again) a versatile, efficient and state-of-the-art tool developed by the Kelley lab. Overall, the presentation of the methodology is clear and the benchmarks clearly show the high accuracy and strength of this method. Both a web-version and source code are available offering access to biologist and data scientists. Below a set of mainly minor comments.

Response 1
We thank the reviewer for the supportive comments and have addressed the issues they have raised in the point by point responses below.

Comment 2
Page 2, line 36. This paragraph ends with FASTA and BLAST, why are DIAMOND and MMSEQS2 not mentioned here? Currently, this paragraph is not giving an up-to-date overview of the important sequence similarity methods used in bioinformatics.

Response 2
We thank the reviewer for the comment, we have now extended this paragraph to include the suggested methods to bring it up to the present day.

Comment 3
Page 2, line 47: in case tools like eggNOG-mapper (https://pubmed.ncbi.nlm.nih.gov/34597405/ ) or TRAPID (https://pubmed.ncbi.nlm.nih.gov/34197621/) offer similar functionality, please add these and/or explain how SHOOT is different.

Response 3
We thank the reviewer for these suggestions. These tools provide functional annotation based on pre-computed gene families, we have added descriptions to the manuscript.

Comment 4
Page 3, line 71. When searching a sequence against the pre-computed homology groups, is there always a clear top-match, with superior score to the second-best homology group? What happens in case the two top matches have identical scores?

Response 4
We thank the reviewer for this suggestion. To address this possibility, we have added functionality to infer both (or multiple) trees when there are other potential good matches. To be conservative, we have used a wide margin such that we consider an assignment to another tree as possible if the e-value for its hit is less than 1010 times greater than the e-value of the best hit. In such cases we report the e-values for each of the possible assignments and the corresponding trees. This has been described in the manuscript.

Comment 5
(major) Page 5 and Figure 1: how does the speed and accuracy of SHOOT compare to a DIAMOND best-hit approach?

Response 5
We have now added this.

Comment 6
Output SHOOT: a tab-delimited "species /t gene_id /t sequence" output file would be useful to integrate SHOOT in data analysis workflows (when running locally). Is this possible? A related minor comment, did the authors consider offering SHOOT through an API?

Response 6
The returning of data in a format that can be used by workflows is a good point. Regarding the suggestion of "species /t gene_id /t sequence": We return the requested data but in FASTA format instead. While the species vs sequence IDs must be extracted from this format by splitting on the 2nd underscore rather than a tab as proposed by the Reviewer, the ubiquity of the FASTA format means this should fit in better to other workflows. The format is very similar to the suggestion: “genus_species_gene\nsequence”. The format “speciesStr1_speciesStr2_gene” is guaranteed by SHOOT to allow programmatic use.
Regarding an API, we have considered this option and is something we hope to be able to provide in the future. However, we currently cannot currently provide the computational resources necessary to support an API interface. This is something that we hope to develop in a future iteration of SHOOT should funding applications be successful. Although we cannot currently provide an API for SHOOT we do provide the command line program for such expert users.

Comment 7
Tool and website (www.shoot.bio). The website does not mention under which license this tool is released. Furthermore, the Methods section mentions the source code is available (via git) to run this on local hardware, please add this info to the SHOOT website (FAQ).

Response 7
We thank the reviewer for identifying this. The website now states on each page that it is provided under a Creative Commons by attribution licence. The FAQ also describe this. The FAQ also inform the user of the command line tool and provides the github link. The new information has been added to the methods section of the manuscript.

Comment 8
Page 9, line 208 "While, SHOOT identifies" rephrase

Response 8
Done

Comment 9
Page 10, line 244 "The advantage of the creating complete homologous groups" rephrase.

Response 9
Done

Comment 10
Suppl. Info: Several of the tables lack (Uniprot proteome) identifiers to indicate which genome annotation was used. Please add this info so users know which genome assembly/annotation version is integrated in the reference databases.

Response 10
Done

**Second round of review**

**Reviewer 1**

This version address all my questions.

**Reviewer 2**

I thank the authors for the revised manuscript and for performing the additional requested experiments.

Minor comments:
Page 5, first line "The mean time for a complete a SHOOT search of a database" revise
Figure 2: Y-axis: the label "MAP@k" is very cryptic, consider renaming to "Mean Average Precision at k (MAP@k)"
